# Diffuse Large B-Cell Lymphoma, Not Otherwise Specified (DLBCL NOS) Presenting as Multiple Subcutaneous Nodules: An Unusual Cutaneous Presentation of Systemic Disease

**DOI:** 10.1155/2023/2960965

**Published:** 2023-10-03

**Authors:** Nika Tavberidze, Daniel D. Bennett, Daniel R. Matson

**Affiliations:** ^1^Departement of Pathology and Laboratory Medicine, University of Wisconsin-Madison, Madison, Wisconsin, USA; ^2^Department of Dermatology, University of Wisconsin-Madison, Madison, WI, USA

## Abstract

Diffuse large B-cell lymphoma, not otherwise specified (DLBCL NOS) is the most common lymphoid malignancy in the Western world and classically presents as a rapidly enlarging nodal or extranodal mass. Cutaneous involvement by systemic DLBCL NOS is an infrequent clinical presentation, encountered in only 1.5-3.5% of cases, while disseminated cutaneous disease with multiple subcutaneous nodules at the time of diagnosis is unusual and can present a diagnostic challenge. The differential diagnosis when encountering a high-grade B-cell malignancy at a cutaneous site is broad and includes primary cutaneous follicle center lymphoma (PCFCL), primary cutaneous diffuse large B-cell lymphoma, leg type (PCDLBCL-LT), high-grade B-cell lymphoma with *MYC* and *BCL2* rearrangements (HGBCL-*MYC/BCL2*), and other potential entities which must all be carefully considered before rendering a final diagnosis. In this report, we describe the case of a 69-year-old man who was seen at our hospital due to generalized weakness and was found to have multiple subcutaneous nodules representing disseminated DLBCL NOS. The case was complicated by concurrent monoclonal B-cell lymphocytosis involving the bone marrow.

## 1. Introduction

Skin manifestations can represent the initial clinical presentation for both T- and B-cell neoplasms. By far, primary cutaneous T-cell lymphomas are the most common hematolymphoid neoplasms of cutaneous origin, accounting for 75% of all primary cutaneous lymphoma cases [1, 2]. The remaining 25% mostly represent PCFCLs, primary cutaneous marginal zone lymphomas, and PCDLBCL-LTs. While rare, DLBCL NOS can also manifest as an isolated cutaneous disease. Identifying and diagnosing DLBCL NOS in this setting can be challenging and require clinicians to consider more common alternative diagnoses, such as PCFCL, PCDLBCL-LT, HGBCL-*MYC/BLC2*, B-lymphocytic leukemia/lymphoma (B-ALL), and mantle cell lymphoma (MCL) [3, 4]. As the clinical behavior and treatment of DLBCL NOS are distinct from these entities, an accurate diagnosis is critical to ensuring appropriate patient management and an optimal outcome [2].

## 2. Case Presentation

**Figure 1:**
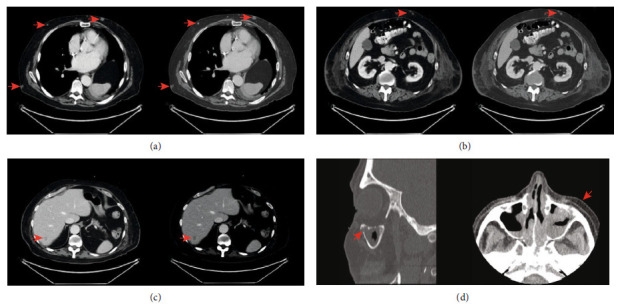
CT scan identified no significant lymphadenopathy but revealed numerous subcutaneous soft tissue nodules throughout the (a) chest and (b) abdominal wall. (c) A low-attenuation hepatic lesion within segment VII of the liver. (d) A sagittal and transverse view of maxillary sinuses shows bilateral mucosal thickening and focal erosion of the anterior wall of the left maxillary sinus with extrasinusoidal extension into the overlying soft tissue. Note: red arrows indicate relevant findings.

A 69-year-old man with a history of polymyalgia rheumatica managed with low-dose glucocorticoid therapy, chronic rhinosinusitis, and hypertensive cardiovascular disease presented to the emergency department with a subacute progressive worsening of generalized weakness. On examination, the patient had a significant truncal weakness with sitting up and rolling over in bed despite showing a full range of motion of all extremities. Palpation of the skin revealed multiple soft subcutaneous nodules on the upper extremities, abdomen, chest, and flanks. Vital signs and physical examinations were otherwise within normal limits. A complete blood count with differential was significant for macrocytic anemia with Hg of 8.7 g/dL (ref. range 13.6-17.2 g/dL) and MCV of 101 fL (ref. range 80-97 fL), thrombocytopenia with a platelet count of 91 K/*μ*L (ref. range 160-370 K/*μ*L), and mild leukocytosis with a WBC of 11.0 K/*μ*L (ref. range 3.5-10.5 K/*μ*L). Circulating absolute lymphocyte count was normal at 1,220 K/*μ*L (ref. range 1,000-3,500 K/*μ*L). Computed tomography including the head, neck, chest, abdomen, and pelvis revealed disseminated indeterminate subcutaneous soft tissue nodules and a low-attenuation hepatic lesion (Figure 1). Additionally, there was bilateral maxillary sinus mucosal thickening with focal bone erosion and extension into surrounding soft tissue. A subcutaneous nodule from the central abdomen was sampled by punch biopsy. The bone marrow and maxillary sinus mucosa were also biopsied.

**Figure 2:**
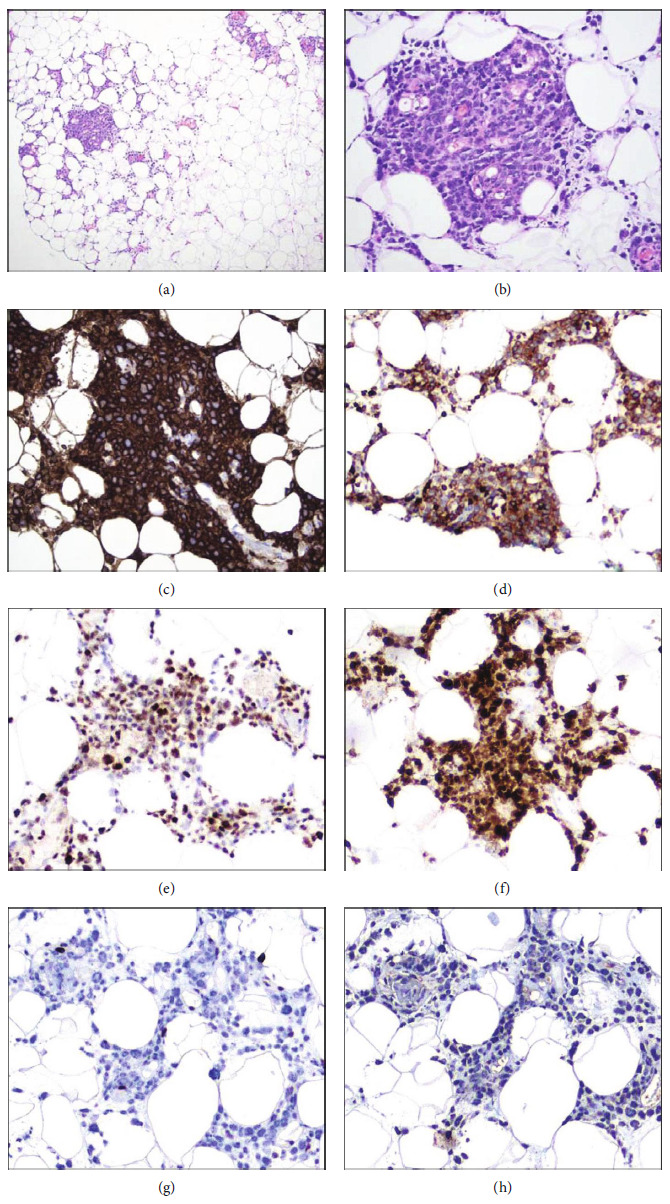
Deep portion of cutaneous bunch biopsy. (a, b) Loose aggregates of large malignant hematolymphoid cells within subcutaneous adipose tissue (H&E, ×100 and ×400). The malignant cells show strong immunoreactivity for (c) CD20, (d) CD5, (e) BCL6, and (f) MUM1 and were negative for (g) CyclinD1 and (h) TdT (DAB with hematoxylin counterstain, ×400).

**Figure 3:**
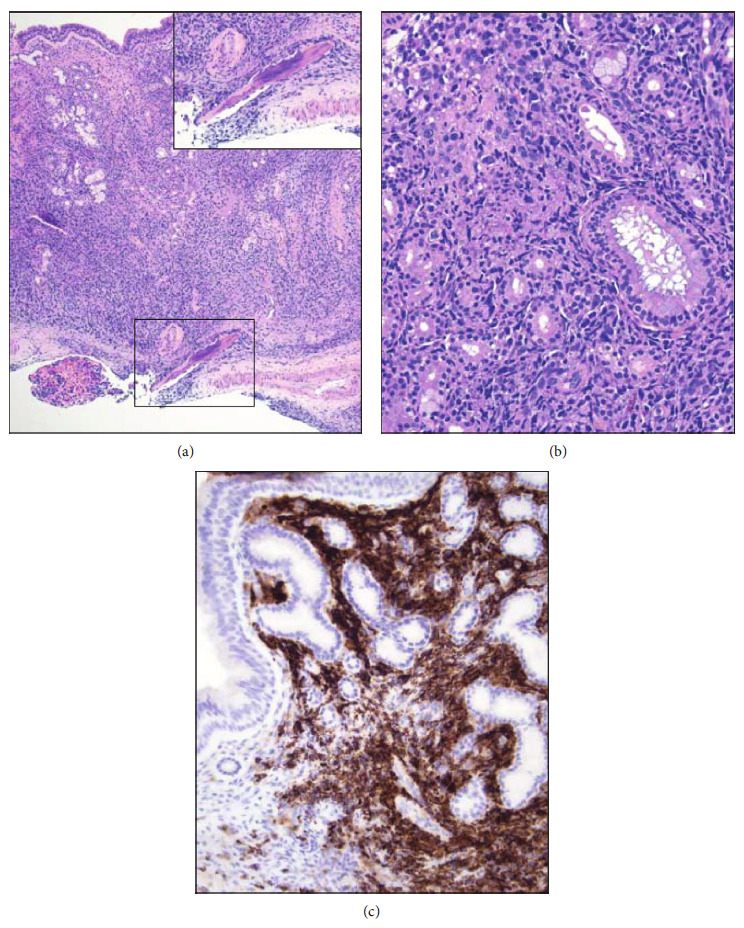
Maxillary sinus biopsy. (a) Dense mucosal and submucosal infiltrates of malignant hematolymphoid cells which extend to underlying bone (inset and white arrow) (H&E, ×100). (b) Involvement and destruction of adnexal structures (H&E, ×400). (c) The malignant cells show strong CD20 immunoreactivity (DAB with hematoxylin counterstain, ×400).

A light microscopic examination of the subcutaneous abdominal nodule revealed mostly unremarkable epidermis, dermis, and subcutis. However, there was a focal aggregate of large atypical hematolymphoid cells within one fragment of deep adipose tissue (Figures 2(a) and 2(b)). Immunohistochemistry (IHC) performed for CD3, CD10, CD20, CD45, CD56, BCL2, CyclinD1, MUM1/IRF4, MYC, p63, PAX5, SOX10, SOX11, and TdT revealed strong and uniform staining for CD20 (Figure 2(c)), CD45, CD5 (Figure 2(d)), BCL2, BCL6 (Figure 2(e)), MUM1/IRF4 (Figure 2(f)), and PAX5. The cells were negative for all other markers (Figures 2(g) and 2(h)). Epstein-Barr virus in situ hybridization was negative. An identical population of malignant cells was found to be infiltrating separately submitted fragments of the sinus mucosa (Figure 3).

**Figure 4:**
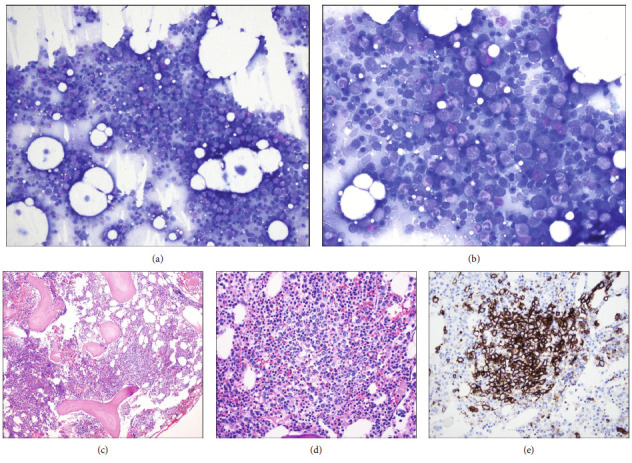
Bone marrow biopsy. (a, b) Bone marrow aspirates showing rare scattered atypical hematolymphoid cells (white arrow) with large nuclei and prominent nucleoli (Wright-Giemsa, ×200 and ×400). (c, d) Bone marrow core biopsy demonstrating loose lymphoid aggregates (H&E, ×100 and ×400) highlighted by (e) strong CD20 immunoreactivity (DAB with hematoxylin counterstain, ×400).

**Figure 5:**
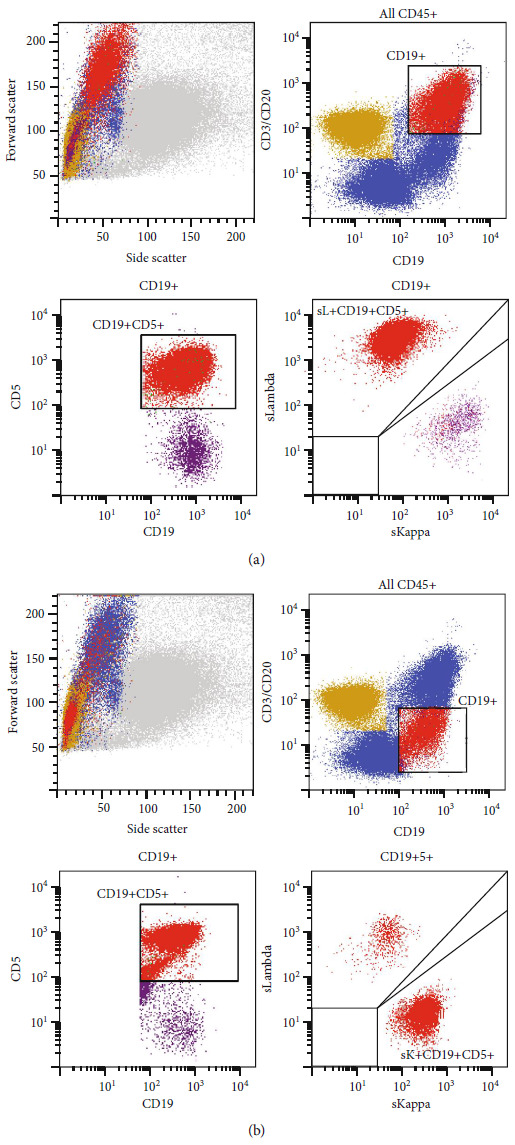
Two distinct populations of abnormal B-cells were identified on flow cytometric evaluation of the bone marrow. (a) One population of lambda light chain-restricted CD5-positive B-cells, which appear large on forward scatter, representing the malignant lymphocytes of DLBCL NOS (highlighted in red). (b) A second incidental population of uncertain significance composed of kappa light chain-restricted CD5-positive B-cells that appear of similar size as background lymphocytes by forward scatter and show dim expression of light chain and CD20 (highlighted in red).

Bone marrow aspirate revealed scattered large atypical hematolymphoid cells in a background of otherwise morphologically normal trilineage hematopoiesis (Figures 4(a) and 4(b)). The bone marrow core biopsy confirmed loose aggregates of large atypical lymphoid cells with strong CD20 immunoreactivity (Figures 4(c)–4(e)). Interestingly, concurrent flow cytometry of bone marrow revealed two distinct abnormal B-cell populations: (1) a population of large lambda light chain-restricted CD5-positive B-cells, consistent with the atypical lymphoid cells noted in the aspirate (Figure 5(a)), and (2) a unique population of kappa light chain-restricted CD5-positive B-cells with a CD20-dim, light chain-dim, CD23-positive, CD200-positive immunophenotype diagnostic of chronic lymphocytic leukemia (CLL) (Figure 5(b)). The second B-cell population was most consistent with monoclonal B-cell lymphocytosis per the World Health Organization (WHO) Classification of Haematolymphoid Tumors and was favored to represent an incidental finding of unclear clinical significance.

The differential diagnoses of a primarily cutaneous high-grade B-cell neoplasm with CD5 expression included PCDLBCL-LT, HGBCL-*MYC/BCL2*, MCL, DLBCL NOS, and B-ALL (Table 1). To evaluate these possibilities, fluorescent in situ hybridization (FISH) for *MYC* gene rearrangements and the *MYC*/*IgH* fusion were performed, which was negative. This result ruled out HGBCL-*MYC*/BCL2. The diagnosis of PCDLBCL-LT was considered unlikely given the absence of lower extremity involvement, the male sex of the patient, the lack of a *MYC* translocation, and the absence of monotonous morphology. Negative IHC for TdT ruled out B-ALL, and CyclinD1 and SOX11 were both negative for IHC, which ruled out MCL. The morphologic, immunophenotypic, and clinical features of the neoplasm were all consistent with a DLBCL NOS, and cell-of-origin subtyping yielded an activated B-cell (ABC) subtype per the Hans criteria.

The diagnosis and treatment options were discussed with the patient, who opted for palliative care and died 22 days after the initial presentation.

## 3. Discussion

DLBCL NOS is the most common non-Hodgkin lymphoma in Western countries, accounting for 31% of adult cases [5]. Patients may present with constitutional B-symptoms (fever, night sweats, and weight loss) and/or a rapidly enlarging mass at nodal or extranodal sites [6, 7]. Disseminated disease to the bone marrow is seen in 20% of patients at diagnosis and can represent either DLBCL NOS (concordant involvement) or indolent lymphoma (discordant involvement) [8–10]. DLBCLs are phenotypically and genetically heterogeneous and are typically broadly divided into transcriptionally defined germinal center B-cell (GCB) and ABC subtypes based on cell-of-origin, with an ABC subtype conferring an adverse prognosis [11–13]. Different IHC algorithms have been developed for routine cell-of-origin subtyping. The most widely used is the Hans algorithm which scores expression of CD10, BCL6, and MUM1/IRF4 [13, 14].

Cutaneous presentations by DLBCL NOS occur in only 1.5-3.5% of cases [15, 16]. High-grade B-cell lymphoma, when encountered in the skin, raises several diagnostic considerations including PCFCL, PCDLBCL-LT, HGBCL-*MYC/BCL2*, and B-ALL. When positive for CD5, the differential also includes MCL (Table 1). These entities can typically be assessed via a thorough review of the clinical history and physical exam, coupled with a careful evaluation of the tissue pathology (including IHC, cytogenetics, and/or molecular testing as necessary).

PCFCL is a tumor of neoplastic follicle center B-cells that accounts for 50% of primary cutaneous B-cell lymphomas and characteristically presents as skin lesions on the head, neck, or trunk [17]. However, 15% show multifocal skin involvement. Cells appear as a mixture of centrocytes and centroblasts which may show follicular, follicular and diffuse, or diffuse architecture, usually with epidermal sparing [1]. PCFCLs are positive for the germinal center marker CD10. However, unlike nodal follicle center lymphomas, PCFCLs are negative for BCL2 [18]. When follicular architecture is not obvious, CD21 may help by highlighting associated follicular dendritic cell meshworks. When only a diffuse architecture with monotonous populations of centroblasts is present, a diagnosis of PCDLBCL-LT should be strongly considered [1]. PCFCLs have an excellent prognosis, with a 5-year survival rate greater than 95% [2]. Our patient's lymphoma was negative for CD10, had high-grade morphology, and showed aggressive behavior with involvement of the bone marrow, which was all less consistent with PCFCL.

PCDLBCL-LT accounts for 10-20% of primary cutaneous B-cell lymphomas and is typically present on the skin of the lower extremities in elderly women (female : male ratio of 2-4 : 1) [19, 20]. Progression to disseminated disease with involvement of extracutaneous sites is common, but usually absent at diagnosis. It appears as a monotonous infiltrate in the dermis and subcutis with frequent mitotic figures and epidermal sparing [19]. The cells are positive for BCL2, MUM1/IRF4, FOXP1, MYC, and IgM, while CD10 is typically negative. The 5-year survival rate is 50%; however, recent studies incorporating rituximab have reported improved outcomes [19, 21].

HGBCL-*MYC*/*BCL2* is a high-grade B-cell lymphoma harboring *MYC* and *BCL2* rearrangements [4]. HGBCL-*MYC*/*BCL2* typically presents as a disseminated disease at diagnosis in elderly individuals with a slight male predominance [22, 23]. Most HGBCL-*MYC*/*BCL2* show morphology that is indistinguishable from DLBCL NOS and are positive for CD10, BCL2, BCL6, and MYC by IHC [13, 24]. Mandatory FISH studies confirm rearrangements involving the *MYC* and *BCL2* genes. HGBCL-*MYC*/*BCL2*s follow an aggressive clinical course, and patients have poor outcomes with 5-survival rates of 40-50% [25].

MCL can display large cell features and should enter the diagnosis when high-grade B-cell lymphomas express CD5 [26]. They are positive for CyclinD1 by IHC, and rare CyclinD1-negative MCLs are positive for SOX11. High-grade variants of MCL have poor clinical outcomes [27]. Finally, extramedullary B-ALL should be considered in unusual presentations of apparent DLBCL NOS [28]. B-ALL expresses TdT and is usually negative for CD20 and the light chain. B-ALL is aggressive, and survival is highly age-dependent, with 20% 5-year overall survival among ages 65-74 (our patient's age group).

In conclusion, we described a patient who presented with a nonspecific symptomatology. Multiple unexplained subcutaneous nodules seen on physical exams and imaging studies prompted the decision to biopsy. Disseminated disease with the absence of lower extremity involvement, high-grade morphology with cellular pleomorphism and diffuse architecture, negative IHC for CyclinD1, SOX11, and TdT, and absence of characteristic cytogenetic rearrangements involving *MYC* and *BCL2*, ultimately yielded a diagnosis of DLBCL NOS.

## Figures and Tables

**Table 1:** Example differential diagnosis of a primarily cutaneous high-grade B-cell neoplasm with CD5 expression.

High-grade CD5+ B-cell lymphoma with primary cutaneous involvement (select diagnostic considerations and WHO 5^th^ edition criteria)
Primary cutaneous follicle center lymphoma Essential Follicular and/or diffuse proliferation of centrocytes and admixed centroblasts B-cells with coexpression of germinal center markers (e.g., CD10 and BCL6) No extracutaneous involvement by lymphoma Desirable Localization to head or trunk Evidence of B-cell monoclonality Absent or weak BCL2 expression (usually) Lack of MUM1 expression Absence of *BCL2* rearrangement (usually)
Primary cutaneous diffuse large B-cell lymphoma, leg type Essential Morphology and phenotype consistent with aggressive B-cell lymphoma Mature B-cell phenotype Diffuse growth with absence of follicular dendritic cell meshworks Skin-confined disease at presentation Desirable Strong BCL2 expression Expression of IgM and MUM1, non-GCB phenotype
High-grade B-cell lymphoma with MYC and BCL2 rearrangements Essential Morphology and phenotype consistent with aggressive B-cell lymphoma Evidence of concurrent *MYC* and *BCL2* rearrangements Desirable GCB Phenotype TdT protein expression status (negative) Determination of *MYC* fusion partner
Mantle cell lymphoma (CD5+) Essential B-cell immunophenotype with CD5 expression Classic or variant (e.g., blastoid or pleomorphic) morphology CyclinD1 positive or detection of *CCND1* rearrangement Desirable SOX11 positive
B-lymphoblastic leukemia/lymphoma (lymphoma-type presentation) Essential Diffuse involvement by a monomorphic population of blasts with a B-cell phenotype (CD19, CD22, cCD79a, and/or PAX5) and markers of immaturity (TdT, CD34, and/or CD10) Desirable Immunophenotype associated with specific recurrent genetic abnormalities Identification of specific recurrent genetic abnormalities

## Data Availability

The radiology images and pathology sections used to support the findings of this study are included within the article.
